# Sex Hormone Receptor Expression in Craniopharyngiomas and Association with Tumor Aggressiveness Characteristics

**DOI:** 10.3390/jcm11010281

**Published:** 2022-01-05

**Authors:** Antonio Martínez-Ortega, Álvaro Flores-Martinez, Eva Venegas-Moreno, Elena Dios, Diego Del Can, Eloy Rivas, Ariel Kaen, Eugenio Cárdenas Ruiz-Valdepeñas, Elena Fajardo, Florinda Roldán, Natividad González-Rivera, Rosario Oliva, José Ignacio Fernández-Peña, Alfonso Soto-Moreno, David A. Cano

**Affiliations:** 1 Unidad de Gestión de Endocrinología y Nutrición, Instituto de Biomedicina de Sevilla (IBiS), Hospital Universitario Virgen del Rocío/CSIC/Universidad de Sevilla, 41013 Sevilla, Spain; dr.antmarort@gmail.com (A.M.-O.); alvflomar@gmail.com (Á.F.-M.); evam.venegas.sspa@juntadeandalucia.es (E.V.-M.); elenadiosfuentes@gmail.com (E.D.); djds22@gmail.com (D.D.C.); 2 Unidad de Gestión de Anatomía Patológica, Unidad de Neuropatología, Hospital Universitario Virgen del Rocío, 41013 Sevilla, Spain; eloy.rivas.sspa@juntadeandalucia.es; 3 Servicio de Neurocirugía, Hospital Universitario Virgen del Rocío, 41013 Sevilla, Spain; kaenariel@hotmail.com (A.K.); eugeniocarde@hotmail.com (E.C.R.-V.); 4 Servicio de Radiología, Hospital Universitario Virgen del Rocío, 41013 Sevilla, Spain; elena.fajardo.sspa@juntadeandalucia.es (E.F.); florivict@gmail.com (F.R.); 5 Unidad de Gestión de Endocrinología y Nutrición, Hospital Universitario Virgen Macarena, 41009 Sevilla, Spain; natividad-gonzalez@hotmail.com (N.G.-R.); ros_oliva@hotmail.com (R.O.); 6 Unidad de Gestión de Endocrinología y Nutrición, Hospital de Valme, 41014 Sevilla, Spain; jifernandezpe@gmail.com

**Keywords:** craniopharyngiomas, estrogen receptor, progesterone receptor, β-catenin, immunohistochemistry

## Abstract

Craniopharyngiomas (CPs) are rare tumors of the sellar and suprasellar regions of embryonic origin. The primary treatment for CPs is surgery but it is often unsuccessful. Although CPs are considered benign tumors, they display a relatively high recurrence rate that might compromise quality of life. Previous studies have reported that CPs express sex hormone receptors, including estrogen and progesterone receptors. Here, we systematically analyzed estrogen receptor α (ERα) and progesterone receptor (PR) expression by immunohistochemistry in a well-characterized series of patients with CP (*n* = 41) and analyzed their potential association with tumor aggressiveness features. A substantial proportion of CPs displayed a marked expression of PR. However, most CPs expressed low levels of ERα. No major association between PR and ERα expression and clinical aggressiveness features was observed in CPs. Additionally, in our series, β-catenin accumulation was not related to tumor recurrence.

## 1. Introduction

Craniopharyngiomas (CPs) are rare tumors of the sellar and suprasellar regions of embryonic origin that seems to arise from the Rathke’s pouch [1,2]. CPs represent around 1.2–4.6% of all intracranial tumors and their incidence has been estimated to be 0.5–2 cases per million people per year [2]. Although CPs may be diagnosed at any age, 30–50% of the cases are found during childhood and adolescence [3]. There are two CP types that display different clinical, histological, and molecular characteristics [2]. Thus, adamantinomatous CPs constitute about 85–90% of CPs and are usually caused by mutations in the *CTNNB1* gene (which encodes the WNT-mediator protein β-catenin) [4]. Papillary CPs are mostly found in adults and are associated with mutations in BRAF [5].

Although CPs are considered benign tumors and typically display slow growth they are associated with an impaired quality of life due to the tumor’s anatomical localization and/or surgical treatment [1]. Surgery is the first line of treatment for CPs [6,7]. However, CPs are prone to local invasion and adherence to adjacent neurovascular tissues and thus, total removal of the tumors is frequently incomplete [7,8]. Furthermore, surgery might result in significant comorbidities particularly if gross total removal is attempted [8,9]. All of these factors contribute to the increased morbidity and overall mortality seen in CP patients [1,8,10].

Despite their histologically benign nature, CPs display a relatively high recurrence rate [8,11]. In a recent meta-analysis of adult patients with CP, the average recurrence rate was 23% when total resection was achieved and 45% with subtotal resection [11]. Consequently, a great effort has been made to identify predictors of recurrence in CPs [12]. In particular, several histological and molecular biomarkers have been analyzed for their potential association with recurrence and other aggressive characteristics of CPs [13,14]. Nevertheless, none of them has yet achieved the consensus necessary to be used in clinical practice as prognostic factors. Ki-67 is likely the most studied molecular marker regarding CP recurrence but discordant results have been reported [14]. The role of the Wnt signaling pathway in CPs has received considerable attention in recent years. The discovery that mutations in the *CTNNB1* gene that increase the stability of the β-catenin protein are associated with the development of adamantinomatous CPs has prompted the evaluation of β-catenin accumulation as a prognostic factor. Under normal conditions, β-catenin is mostly localized in the cell membrane, but upon activation (for example, due to a mutation), β-catenin is located in the cytoplasm and nucleus. Activation of the Wnt/β-catenin pathway, measured as nuclear and/or cytoplasmic β-catenin localization, has been reported to be associated with CP recurrence [15,16].

CPs have been reported to express sex hormone receptors [17,18]. Interestingly, a relationship between the expression of sex hormone receptors and tumor biological behavior has been described in several intracranial tumors, including meningiomas [19] and pituitary tumors [20,21]. Thus, low levels of estrogen receptor α (ERα) were found to be associated with surgical reintervention in non-functioning pituitary adenomas [21] and an overall worse prognosis in prolactinomas [20]. However, the potential association between sex hormone receptor expression and aggressiveness in CPs has been barely analyzed. One study reported increased recurrence in CPs negative for both estrogen and progesterone receptor (PR) [22] but these results were not confirmed in another study [23]. Based on case reports of women that experienced increased tumor growth during pregnancy, it has been suggested that sex hormones might influence CP behavior [22]. Therefore, this raises the question as to whether CPs expressing high or low levels of their receptors might display different tumor behavior.

The aim of this study was to analyze ERα and PR expression in CPs and determine their potential association with aggressive tumor features.

## 2. Material and Methods

### 2.1. Patients and Samples

Patients who underwent surgery for CPs at the Virgen del Rocío University Hospital (Seville, Spain) between 2001 and 2017 were evaluated retrospectively. The diagnosis of CPs was confirmed histologically by an experienced pathologist (E.R) rechecking the hematoxylin/eosin-stained sections. Only CP samples containing enough tumor tissue and of adequate quality for immunohistochemistry were included. In addition, only patients with a follow-up of at least 3 years were included since this is the reported average time for CP recurrence [12]. Gross total removal was defined as the absence of tumors on postoperative neuroimaging. Recurrence was defined as the detection of a new lesion upon gross total removal or the regrowth of tumor remnant on follow-up MRI neuroimaging. The study complies with the principles of the Helsinki Declaration of the World Medical Association regarding human experimentation and was approved by the IBiS-Virgen del Rocío Hospital Ethics Committee. Biopsy tumor samples were collected and managed by the biobank of the public health system of Andalusia, Spain (Seville Node). 

### 2.2. Histopathology and Immunohistochemistry

Tissue microarrays (TMAs) were generated from archival formalin-fixed paraffin-embedded tissues of CPs. Cores of tissues were obtained from regions of the paraffin blocks identified as tumoral tissue by an experienced pathologist (E.R.) upon inspection of hematoxylin/eosin-stained sections. Duplicates of each CP and samples of normal pituitary tissue were included in TMAs. Immunohistochemical analysis was performed as previously described [24]. Briefly, 5 μm sections of TMAs were deparaffinized, rehydrated, and performed epitope retrieval (Tris-EDTA buffer, pH 9) using the PT Link system (Agilent, Santa Clara, CA, USA). Sections were blocked with 3% donkey serum in PBS for 45 min at room temperature. The following antibodies were used: CONFIRM anti-progesterone receptor (1E2) rabbit monoclonal (Roche, prediluted), one-hour incubation; CONFIRM anti-estrogen receptor (SP1) rabbit monoclonal (Roche, prediluted), overnight incubation; and β-catenin mouse monoclonal (Cell Marque, 224M-17, prediluted), one-hour incubation. Diaminobenzidine (DAB) visualization was performed with the Envision Dual Link System-HRP DAB+ Kit (Agilent, Santa Clara, CA, USA) according to the manufacturer’s instructions. As a technical control, ERα and PR expression were analyzed in twenty-year old paraffin-embedded tissue from breast tumors and found to work properly. Ki-67 immunohistochemistry (clone 30-9, VENTANA, Roche, catalog number 790-4286), was performed using an automated immunostainer system (VENTANA, Roche, Basel, Switzerland) following the manufacturer’s instructions. Counterstaining with hematoxylin was also applied. Immunohistochemistry for estrogen receptor β was not performed since we were not able to obtain reliable immunoreactivity with available commercial antibodies. The percentage of Ki-67 positively-stained tumor cell nuclei was used to determine the Ki-67 index. For each sample, three randomly selected high-magnification fields were examined (at least 500 tumor cells were counted). Immunoreactivity for ERα and PR was measured quantitatively using the immunoreactivity score (IRS), a commonly used score for immunohistochemistry in biomedical research [25]. Briefly, IRS is determined as the product of the percentage of positive cells (0 = 0%; 1 = 1–10%; 2 = 11–50%; 3 = 51–79%; and 4 = >80%) and the intensity of immunostaining (0 = no staining; 1 = weak; 2 = moderate; and 3 = strong). Of note was that only nuclear staining for these markers was considered for the scoring. A classification was used to categorize IRS values into four categories: negative (IRS, 0–1); low (IRS, 2–3); medium (IRS, 4–8); and high (IRS, 9–12), as previously described [20]. Scoring for β-catenin was performed as previously described [16]. In short, if the percentage of cells with cytoplasmic and/or nuclear β-catenin expression was greater than 10%, the expression of β-catenin was classified as “aberrant”. Otherwise, the expression of β-catenin was categorized as “preserved”. Both duplicates of each CP in the TMA were scored. If there was disagreement between the scores, the highest value was selected.

### 2.3. Statistical Analysis

The normality of the data was assessed using the Kolmogorov–Smirnov test. Categorical variables are described as percentages and frequencies. Non-normally distributed data are indicated as median values and interquartile ranges (IQR). For normally distributed data, mean and SD are used. ANOVA and Student’s t tests were used for parametric variables and Kruskal–Wallis and Mann–Whitney tests for nonparametric variables. Chi-square was used for categorical variables. Spearman’s rank correlation coefficient was used for correlation analysis between continuous variables. *P* values were adjusted for multiple comparisons by the Benjamini–Hochberg false discovery rate method. Statistical analysis was performed using SPSS software version 25.0 (SPSS, Chicago, IL, USA). *P* values < 0.05 were considered statistically significant.

## 3. Results

### 3.1. Patient and Sample Characteristics

A total of 41 CP samples met the inclusion criteria. The baseline clinical characteristics of the study population are shown in Table 1. Primary tumor tissue was available for 30 samples; in 11 cases, only the recurrent tumor was available. Surgical resection was subtotal (defined as the presence of tumor remnants after surgery) in 73.2% of patients. Tumor relapse (combined tumor regrowth and recurrence events) within three years of follow-up was found in 53.7% of the patients. Of those, only two patients showed recurrence (i.e., tumor formation after gross total removal of the tumor) within three years of follow-up.

### 3.2. Estrogen and Progesterone Receptor Expression in Craniopharyngiomas

**Figure 1:**
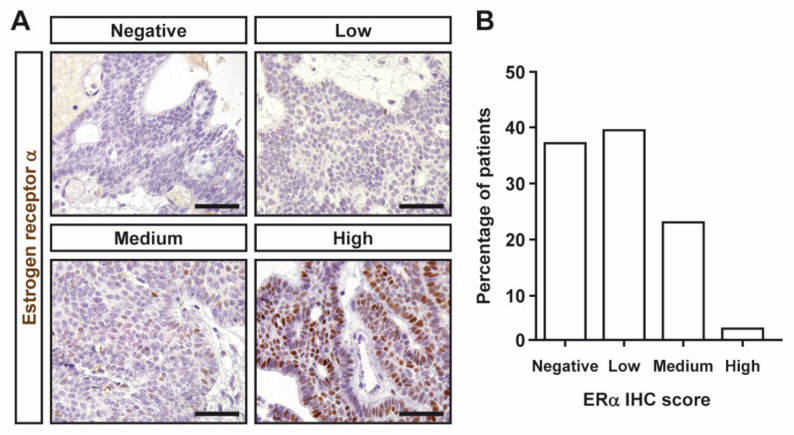
Immunohistochemical detection of estrogen receptor α (ERα) in CPs assessed by immunohistochemistry. (**A**) Representative pictures of ERα immunohistochemical categories in CPs. Negative, no or only cytoplasmic immunoreactivity, IRS 0–1; low, IRS 2–3; medium, IRS 4–8; and high, IRS 9–12. Scale bar: 50 μm. (**B**) Percentage of patients with CPs for IHC categories.

**Figure 2:**
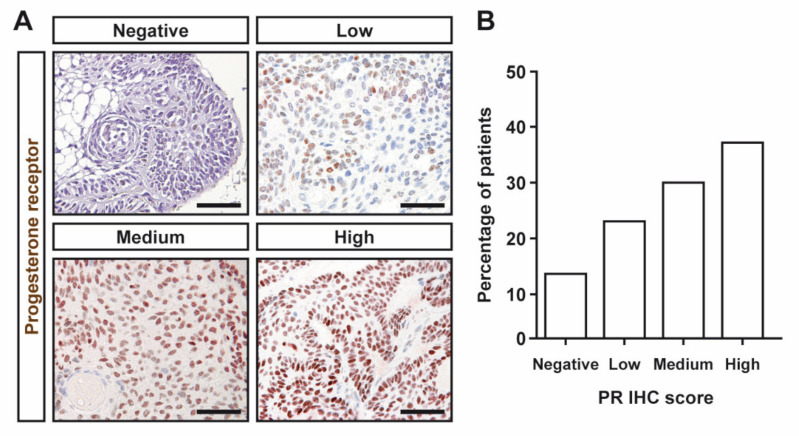
Immunohistochemical detection of progesterone receptor (PR) in CPs assessed by immunohistochemistry. (**A**) Representative pictures of PR immunohistochemical categories in CPs. Negative, no, or only cytoplasmic immunoreactivity, IRS 0–1; low, IRS 2–3; medium, IRS 4–8; and high, IRS 9–12. Scale bar: 50 μm. (**B**) Percentage of patients with CPs for IHC categories.

Most CPs expressed ERα, albeit at low levels. The IRS median was 2 (IQR, 1–3.5). Representative images of ERα immunoreactivity of the different IHC semiquantitative grades in CPs are shown in Figure 1A. Sixteen CPs displayed no or negligible nuclear staining (Figure 1B). Only one tumor showed high ERα levels (Figure 1B). Representative images of PR immunoreactivity of the different IHC semiquantitative grades in CPs are shown in Figure 2A. A high proportion of CPs expressed substantial PR levels (12 and 15 CPs were classified as medium and high categories, respectively). Indeed, only five CPs were found to display any PR immunoreactivity (Figure 2B). The IRS median was 6 (IQR, 2–9). No differences in ERα or PR expression (as assessed by both IRS and semiquantitative categories) were found between males and females. Additionally, no correlation between ERα and PR expression was observed.

### 3.3. Association between Estrogen and Progesterone Receptor Levels and Clinical Features of Craniopharyngiomas

We assessed the potential association between ERα or PR expression levels and major features of aggressiveness in CPs, namely tumor relapse. No significant differences were found between tumor relapse within three years of follow-up and expression levels of ERα or PR, as assessed by both the IRS and semiquantitative categories. We performed the same analysis in males and females separately but CPs with or without relapse did not display significantly different IRS values of ERα or PR.

Regarding other clinical characteristics, no significant correlations between ERα and PR expression and age at diagnosis were observed. Similarly, ERα and PR expression was not significantly different between pediatric and adult patients. No significant correlation between ERα expression and tumor size was found. However, expression levels of PR (as assessed by IRS) were directly correlated to tumor size (r = 0.497, Spearman FDR adjusted *p* = 0.02). Indeed, tumor size was increased in CPs with high PR expression compared to CPs with low PR expression (*p* = 0.005). Almost half of CPs (46.3%) displayed high Ki-67 levels (>3%). The median Ki-67 index for CPs was 2.5 (IQR, 0.89–5.22). However, no correlation between Ki-67 levels and ERα or PR IRS scores was found. Of note is that we did not find differences between Ki-67 levels and tumor relapse. Finally, we did not find statistically significant differences in expression levels of ERα or PR (as assessed by IRS) between CP histopathologic types, although we have to note that the number of papillary CPs in our cohort was low (five). Two papillary CPs showed high PR levels, two showed low levels, and one was negative. Regarding ERα, one papillary CP displayed medium levels, three displayed low levels, and one was negative.

### 3.4. β-Catenin Expression and CP Recurrence

**Figure 3:**
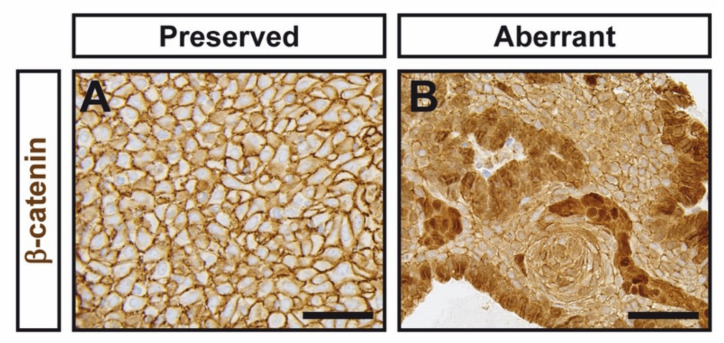
Immunohistochemical detection of β-catenin in CPs assessed by immunohistochemistry. (**A**) Representative picture of a CP showing preserved β-catenin accumulation. (**B**) Representative picture of a CP showing aberrant β-catenin accumulation. Scale bar: 50 μm.

Since previous studies have suggested an association between nuclear and/or cytoplasmic β-catenin accumulation and the risk of recurrence in CP [15,16] we sought to investigate the association of β-catenin accumulation with aggressive features in our series of CPs. Since papillary CPs are not driven by the Wnt pathway, they were excluded from this analysis. Sixteen (44%) CPs displayed aberrant β-catenin accumulation, as assessed by a previously described scoring system [16]. Representative images of β-catenin immunohistochemistry are shown in Figure 3. However, we did not find statistically significant differences in relapse rates between CPs with aberrant and preserved β-catenin localization. Additionally, no difference was found between β-catenin localization and sex, age, tumor size, or Ki-67 levels.

## 4. Discussion

In this study, we examined the expression of ERα and PR in CP biopsies from 41 patients and analyzed the potential association between their expression and clinical and pathologic features of CPs.

To assess the expression of ERα and PR, we used immunohistochemical methods using reliable, diagnostic-grade antibodies. Immunohistochemistry has certain advantages over other methods of gene expression analysis, such as quantitative real-time PCR. Although immunohistochemistry is not a complete quantitative method to measure protein accumulation, it allows precise protein localization within tissues. This is particularly important in tumors such as CPs, which can have a high amount of stromal tissue surrounding the tumoral cells. Thus, immunohistochemistry allows the quantification of protein accumulation specifically in tumoral cells and, therefore, provides a more accurate scoring. In our study, we measured immunoreactivity for ERα and PR using the immunoreactivity score (IRS). We decided to implement this scoring method since it is a widely used system for immunohistochemistry in biomedical research [25]. This method also allows us to obtain a semiquantitative measure of protein expression. Indeed, this scoring system has been previously used to evaluate ERα and PR levels in other types of intracranial tumors [21]. We observed that the majority of CPs (75.6%) displayed negative or low ERα expression levels. Furthermore, only one CP showed elevated ERα levels. On the contrary, most CPs (65.9%) exhibited medium or high PR expression levels. Sex hormone receptor expression in CPs has been scarcely analyzed [17,18,22,23]. Nevertheless, our findings are largely in agreement with results from previous studies that also used immunohistochemistry (although antibodies were obtained from a different vendor) to evaluate ERα and PR expression in CPs [23]. Sex differences in ERα or PR expression have been reported in other types of tumors. However, in line with previous studies [17,18,23], we did not find significant differences in ERα or PR expression between sexes.

It has been postulated that sex hormones may impact CP behavior [22] based on case reports of women who experienced accelerated tumor growth during pregnancy [26,27]. However, we found no association between ERα and PR expression and tumor relapse, results that are in agreement with a previous study [23]. Another study found an increased risk of recurrence in CPs with negative expression of both ERα and PR [22]. We were not able to test this observation since the number of CPs negative for both receptors in our study was too low to make meaningful analyses. Regarding other clinical variables, we found a positive correlation between PR expression and tumor size. However, the biological relevance of this finding it is unclear since PR expression was not associated with proliferation levels (as assessed by Ki-67 immunohistochemistry). Similarly, ERα expression did not show any association with Ki-67 levels. Finally, ERα and PR expression were not associated with age at diagnosis. Since previous studies found that the association between ERα expression and prognosis in pituitary tumors (and other tumor types [28]) was sex-related [21], we performed subgroup analysis by sex. Again, no association between ERα and PR expression and risk of recurrence was observed. Altogether, our results indicate that ERα and PR expression are not associated with major aggressive tumor features, results largely in line with a previous study that analyzed ERα and PR expression by quantitative real time PCR [23]. An association between ERβ expression and tumor biological behavior has been described in pituitary tumors [29]. It would be interesting to analyze ERβ expression in CPs. However, we were not able to find a reliable commercial ERβ antibody and thus its expression could not be analyzed in our study.

Aberrant β-catenin cellular localization has been described as associated with CP recurrence [15,16]. We analyzed β-catenin accumulation using the dichotomous classification previously described by Li et al. [16] in our series of CPs. A substantial proportion of CPs (44.4%) displayed aberrant β-catenin accumulation, i.e., more than 70% of cells showed nuclear and/or cytoplasmic localization. The proportion of CPs with aberrant β-catenin accumulation observed in our cohort is fully in line with that reported by Li et al. [16]. However, in contrast to previous studies [15,16], we found no association between abnormal β-catenin accumulation and tumor relapse. Nevertheless, we need to note that the follow-up in our cohort was restricted to three years while the follow-up duration in those studies was longer (for instance, the median duration was 42.5 months in Li et al. [16]), therefore limiting comparison with our study. Additionally, variations in terms of patient cohort might also account for this discrepancy. For purposes of comparison with these previous studies, a longer follow-up in our cohort will be necessary. However, at least in our hands, β-catenin accumulation does not seem to predict short-term tumor recurrence. In line with the literature [15,16] no other correlations between β-catenin accumulation and other clinical variables (sex, age at diagnosis, and tumor size and proliferation levels) were found.

We need to acknowledge several limitations in our study. Some of these limitations are inherent to research in CPs, such as the retrospective design, low prevalence of these tumors, and heterogeneity of the patient cohort (in our case, pediatric and adult patients were combined). Additionally, we need to note that the risk of recurrence in CP is largely related to subtotal removal of the tumor [1,14] and thus surgery success might be a confounding factor in our analysis. Finally, although our study includes a relatively large number of samples to properly assess sex hormone receptor expression, subgroup analysis (for instance, by sex as discussed above) is somewhat hampered.

In conclusion, we found that most CPs express relatively low levels of ERα. By contrast, most CPs express high levels of PR. ERα and PR expression was not associated with the major aggressive features of CPs. Additionally, no significant associations between β-catenin accumulation and major clinical variables of interest were observed in our cohort. Our finding that a considerable proportion of CPs display appreciable levels of ERα expression might have potential therapeutic implications for the use of estrogen receptor inhibitors, such as tamoxifen, for CP treatment as in other types of hormone-dependent tumors.

## Figures and Tables

**Table 1:** Baseline characteristics of the study cohort.

Characteristics	
Sex (% female)	46.3%
Age at diagnosis (years, median, IQR)	30 (9.5–58)
Pediatric patients [younger than 16] years (*n*, %)	15 (36.6%)
Maximum tumor diameter at diagnosis (mm, median, IQR) *	34 (25–40)
Histopathologic type (*n*, %)	
Adamantinomatous	36 (87.8%)
Papillary	5 (12.2%)

* Data were available for only 31 patients.

## Data Availability

The data presented in this study are available on request from the corresponding authors.

## References

[1] Muller H.L. (2014). Craniopharyngioma. Endocr. Rev..

[2] Muller H.L., Merchant T.E., Warmuth-Metz M., Martinez-Barbera J.P., Puget S. (2019). Craniopharyngioma. Nat. Rev. Dis. Primers.

[3] Bunin G.R., Surawicz T.S., Witman P.A., Preston-Martin S., Davis F., Bruner J.M. (1998). The descriptive epidemiology of craniopharyngioma. J. Neurosurg..

[4] Sekine S., Shibata T., Kokubu A., Morishita Y., Noguchi M., Nakanishi Y., Sakamoto M., Hirohashi S. (2002). Craniopharyngiomas of adamantinomatous type harbor beta-catenin gene mutations. Am. J. Pathol..

[5] Brastianos P.K., Taylor-Weiner A., Manley P.E., Jones R.T., Dias-Santagata D., Thorner A.R., Lawrence M.S., Rodriguez F.J., Bernardo L.A., Schubert L. (2014). Exome sequencing identifies BRAF mutations in papillary craniopharyngiomas. Nat. Genet..

[6] Cossu G., Jouanneau E., Cavallo L.M., Elbabaa S.K., Giammattei L., Starnoni D., Barges-Coll J., Cappabianca P., Benes V., Baskaya M.K. (2020). Surgical management of craniopharyngiomas in adult patients: A systematic review and consensus statement on behalf of the EANS skull base section. Acta Neurochir..

[7] Ottenhausen M., Rumalla K., La Corte E., Alalade A., Nair P., Forbes J., Ben Nsir A., Schwartz T.H. (2019). Treatment strategies for craniopharyngiomas. J. Neurosurg. Sci..

[8] Buchfelder M., Schlaffer S.M., Lin F., Kleindienst A. (2013). Surgery for craniopharyngioma. Pituitary.

[9] Apra C., Enachescu C., Lapras V., Raverot G., Jouanneau E. (2019). Is Gross Total Resection Reasonable in Adults with Craniopharyngiomas with Hypothalamic Involvement?. World Neurosurg..

[10] Bulow B., Attewell R., Hagmar L., Malmstrom P., Nordstrom C.H., Erfurth E.M. (1998). Postoperative prognosis in craniopharyngioma with respect to cardiovascular mortality, survival, and tumor recurrence. J. Clin. Endocrinol. Metab..

[11] Dandurand C., Sepehry A.A., Asadi Lari M.H., Akagami R., Gooderham P. (2018). Adult Craniopharyngioma: Case Series, Systematic Review, and Meta-Analysis. Neurosurgery.

[12] Prieto R., Pascual J.M., Subhi-Issa I., Jorquera M., Yus M., Martinez R. (2013). Predictive factors for craniopharyngioma recurrence: A systematic review and illustrative case report of a rapid recurrence. World Neurosurg..

[13] Coury J.R., Davis B.N., Koumas C.P., Manzano G.S., Dehdashti A.R. (2020). Histopathological and molecular predictors of growth patterns and recurrence in craniopharyngiomas: A systematic review. Neurosurg. Rev..

[14] Prieto R., Pascual J.M. (2018). Can tissue biomarkers reliably predict the biological behavior of craniopharyngiomas? A comprehensive overview. Pituitary.

[15] Guadagno E., De Divitiis O., Solari D., Borrelli G., Bracale U.M., Di Somma A., Cappabianca P., Del Basso De Caro M. (2017). Can recurrences be predicted in craniopharyngiomas? Beta-catenin coexisting with stem cells markers and p-ATM in a clinicopathologic study of 45cases. J. Exp. Clin. Cancer Res..

[16] Li Z., Xu J., Huang S., You C. (2015). Aberrant membranous expression of beta-catenin predicts poor prognosis in patients with craniopharyngioma. Ann. Diagn. Pathol..

[17] Honegger J., Renner C., Fahlbusch R., Adams E.F. (1997). Progesterone receptor gene expression in craniopharyngiomas and evidence for biological activity. Neurosurgery.

[18] Thapar K., Stefaneanu L., Kovacs K., Scheithauer B.W., Lloyd R.V., Muller P.J., Laws E.R. (1994). Estrogen receptor gene expression in craniopharyngiomas: An in situ hybridization study. Neurosurgery.

[19] Pravdenkova S., Al-Mefty O., Sawyer J., Husain M. (2006). Progesterone and estrogen receptors: Opposing prognostic indicators in meningiomas. J. Neurosurg..

[20] Delgrange E., Vasiljevic A., Wierinckx A., Francois P., Jouanneau E., Raverot G., Trouillas J. (2015). Expression of estrogen receptor alpha is associated with prolactin pituitary tumor prognosis and supports the sex-related difference in tumor growth. Eur. J. Endocrinol..

[21] Oystese K.A., Casar-Borota O., Normann K.R., Zucknick M., Berg J.P., Bollerslev J. (2017). Estrogen Receptor alpha, a Sex-Dependent Predictor of Aggressiveness in Nonfunctioning Pituitary Adenomas: SSTR and Sex Hormone Receptor Distribution in NFPA. J. Clin. Endocrinol. Metab..

[22] Izumoto S., Suzuki T., Kinoshita M., Hashiba T., Kagawa N., Wada K., Fujimoto Y., Hashimoto N., Saitoh Y., Maruno M. (2005). Immunohistochemical detection of female sex hormone receptors in craniopharyngiomas: Correlation with clinical and histologic features. Surg. Neurol..

[23] Hofmann B.M., Hoelsken A., Fahlbusch R., Blumcke I., Buslei R. (2010). Hormone receptor expression in craniopharyngiomas: A clinicopathological correlation. Neurosurgery.

[24] Venegas-Moreno E., Vazquez-Borrego M.C., Dios E., Gros-Herguido N., Flores-Martinez A., Rivero-Cortes E., Madrazo-Atutxa A., Japon M.A., Luque R.M., Castano J.P. (2018). Association between dopamine and somatostatin receptor expression and pharmacological response to somatostatin analogues in acromegaly. J. Cell Mol. Med..

[25] Meyerholz D.K., Beck A.P. (2018). Principles and approaches for reproducible scoring of tissue stains in research. Lab. Investig..

[26] Magge S.N., Brunt M., Scott R.M. (2001). Craniopharyngioma presenting during pregnancy 4 years after a normal magnetic resonance imaging scan: Case report. Neurosurgery.

[27] Aydin Y., Can S.M., Gulkilik A., Turkmenoglu O., Alatli C., Ziyal I. (1999). Rapid enlargement and recurrence of a preexisting intrasellar craniopharyngioma during the course of two pregnancies. Case report. J. Neurosurg..

[28] Lund-Iversen M., Scott H., Strom E.H., Theiss N., Brustugun O.T., Gronberg B.H. (2018). Expression of Estrogen Receptor-alpha and Survival in Advanced-stage Non-small Cell Lung Cancer. Anticancer Res..

[29] Zhou W., Song Y., Xu H., Zhou K., Zhang W., Chen J., Qin M., Yi H., Gustafsson J.A., Yang H. (2011). In nonfunctional pituitary adenomas, estrogen receptors and slug contribute to development of invasiveness. J. Clin. Endocrinol. Metab..

